# Medicine

**Published:** 1894-03

**Authors:** J. Michell Clarke


### Progress of the Medical Sciences.

### MEDICINE.

Dr. Hale White[1] has been led to make some fresh observations on the influence of diet in Bright's disease, because there is a conflict of opinion as to which is the best diet. A milk diet is frequently recommended on the grounds that it (1) diminishes the amount of albumen, (2) that it is easily digested and absorbed, (3) that it does not irritate the kidneys, (4) that it is diuretic, and (5) that it contains no toxic substances. The author finds, however, that it does not always diminish the albumen, at any rate, in chronic interstitial nephritis; and that even if it does so, there is no proof in many cases that this is of any benefit. The actual amount of albumen passed is rarely of much importance in itself. Many patients with Bright's disease suffer from indigestion when fed solely on milk. It has not been shown that ordinary food does irritate the kidneys. Milk is neither a certain nor a powerful diuretic. The belief that a diet containing very little proteid is beneficial in Bright's disease is probably due to the lingering survival of the false view that retention of urea is the cause of uræmia.

He has made a careful analysis of eight cases of chronic interstitial and two of chronic parenchymatous nephritis. The diets given were: (1) Milk, containing 1076 grains proteid (3 pints milk); (2) Farinaceous, containing 1137 grains proteid (bread 12 oz., milk 41 oz.); and (3) Full diet, containing 1522 grains proteid (bread 12 oz., meat 6 oz., milk rather over 1 pint, and rice-pudding ½-lb., or ½-pint mutton broth).

[1] *Med. Chir. Tr.*, vol. lxxvi.

Dr. Hale White concludes that on the whole an ordinary full diet is best, for (1) it does not increase the liability to uræmia. (2) The general condition is improved upon it; the patients feel stronger and the state of the circulation is better than upon milk or farinaceous diet. (3) There is a saving of albumen to the body, for even if the output of albumen is increased, which is by no means always the case, more than sufficient proteid is taken on the full diet to make up for the extra loss. (4) The effect of diet on urea-excretion is too uncertain to be of any guide, but there is no evidence that full diet is harmful in this respect. (5) There is no evidence that full diet contains or specially leads to the formation of toxic products, which are harmful in chronic Bright's disease. (6) The repugnance felt by these patients to farinaceous diet and to milk is avoided.

On the other hand, less urine is passed upon full than upon the other diets: this may be counterbalanced by the ingestion of more water, and in many cases of chronic Bright's disease diuresis is always profuse. The author thinks that as his 10 cases were sufficiently ill to require admission to hospital, his conclusions might not apply to mild cases or to those persons who are accustomed to overeat themselves.

Dr. Brockbank,[1] in a very valuable and exhaustive paper, states that gall-stones are formed in the following way: The cholesterin is derived from the mucous membrane of the surface of the gall-bladder or from that lining the glands. The shed epithelium falls into bile, which remains an abnormally long time in the gall-bladder. The longer bile remains in the gall-bladder the more cholesterin does it contain. Stagnation of the bile is induced by want of exercise, by an accumulation of fæces in the hepatic flexure of the colon pressing on the cystic duct, by a fundus depressed from distension, or from corset-wearing, until it lies nearly vertically below the outlet of the gall-bladder. When the epithelial cell has remained for some time in the bile, it breaks up into a granular detritus, from which cholesterin crystals subsequently form, or it is transformed in part into "myelin bodies," which turn into crystals. These myelin bodies found in *post-mortem* changes in human bile are of similar nature to those described by the author as formed by the action of soap on cholesterin (see below). If cholesterin is formed as a normal product of the metabolism of the cells, it is secreted in a viscid form, which turns crystalline by combining with the water of the bile. If sufficient bile-salts were present to dissolve the cholesterin as it is formed, no crystals would appear; but when the former are deficient, cholesterin-crystals are deposited and grow into clusters, over which a deposit of cell-detritus, bile pigment, and mucin forms and binds them together. These small masses fall by their weight to the lowest

[1] *Med. Chron.*, Nov., Dec., 1893.

part of the gall-bladder, and remain behind when this is emptied. A concretion tends to increase in bulk by the collection of fresh crystals; when it has reached a certain size, the emptying gall-bladder contracts on it and compresses it into a compact mass. If lime be present in the bile, the deposition of lime-soaps on the concretion will make the mass more stable. The author does not think that the formation of a compound of lime and bile-pigment is essential for the commencement of a calculus, but that the crystals on which the pigment is deposited are cholesterin crystals, though not in the form of plates. Facts which show the influence of diet on formation of gall-stones are: Carnivora do not suffer from gall-stones, except under domestication, when (*e.g.* in the case of pet dogs) sugar, cakes, and carbohydrates enter largely into their diet. This freedom from gall-stones is not connected with the greater quantity of taurocholate of sodium in the bile of carnivora, but with the largely nitrogenous nature of their diet in the free state. Cattle suffer more from gall-stones when stall-fed. In man, Germans suffer more from gall-stones than the English, and the former indulge more freely in breads, carbohydrates, starches, vegetables, and sweets. Gall-stones are more common in the elderly, who take less animal and more carbohydrate foods, and less exercise than younger persons. In 220 livers from diabetic patients, a calculus was only found once (average in all cases, 5-12 per cent.). Gall-stones are frequent in chronic heart-diseases, in which the patients live chiefly on milk and farinaceous diets.

Dr. Brockbank determined the solvent power on gall-stones of various substances in solution in a flask kept at the body-temperature. He found that 1 per cent. solutions of the salicylate, sulphate, benzoate, phosphate, bicarbonate, and chloride of sodium, of phosphate, iodide, bicarbonate, and sulphate of potassium and chloride of ammonium, and 5 per cent. solutions of salicylate and phosphate of sodium and of chloride of ammonium, had no solvent effect. A stone placed in pure olive oil, lost 38 per cent. of its weight in ten days; and in pure oleic acid, a stone after losing 63 per cent. of its weight in two days, broke up into small pieces in four. In a 5 per cent. solution of animal soap in distilled water, the stone became coated after a few hours with a bluish-white filmy structure, together with a club-shaped growth of the same nature springing from one of its edges. Gentle agitation removed this excrescence, which was found to consist of the compound formed by the action of the soap solution on crystals of cholesterin, probably of soap in solution in cholesterin (so-called myelin bodies). In whatever way it is accomplished, the fact remains that a solid has become converted into a viscid substance as the result of the action of soap solution on a gall-stone.

It results that it is useless to administer one of the inorganic cholagogues mentioned above with the idea that they will dissolve a gall-stone; but they are of great use in causing an increased flow of bile, and thus in washing out portions of a gall-stone dissolved by other means. Phosphate or sulphate of sodium are also useful for the same purpose, and further serve as an aperient morning-dose. Belladonna in very large doses is often successful in the treatment of gall-stones, by acting as an antispasmodic. It is generally acknowledged now that cure or improvement frequently follows the administration of olive oil in cases of gall-stones. The author thinks that this is probably due to the antispasmodic action of a large dose of oil, the muscular fibres of the gall-bladder and common duct relaxing, and allowing the stone to escape into the duodenum or to fall back into the bladder. Another explanation he advances from the action of soap and fats on cholesterin. Digested fat passes into the circulation from the intestine as unchanged fat, the corresponding fatty acid, and soap: all occur in normal bile, and the amount present increases with the amount of fat taken in the diet. Oil, fatty acids, and soaps all readily dissolve cholesterin and break up a gall-stone. If then oil, fatty acid, and soap appear in the bile in increased amount after large doses of oil, it is very probable that the gall-stone is attacked by them, and in time dissolved, or reduced in bulk enough to be enabled to be passed out into the duodenum. The objection to the use of olive oil is its extreme unpleasantness when given in large doses of 2—8 oz. One dose in the day is quite sufficient. It may also be injected into the rectum and retained there. From the action of animal soap on gall-stones, the author is inclined to suggest that an increased absorption of animal fat in the form of cream, butter, or fat may be of equal value to olive oil.

The diet should consist of plain food, with abstinence from sugar, starch, and made-up dishes. Alcohol should be taken in the form of light wine only, and never except at meal-times.

Exercise is probably an all-important factor in the treatment of gall-stones.

Dr. A. Lockhart Gillespie[1] in a paper on gastric digestion draws the following conclusions: Free HCl is secreted by the gastric glands from the moment of ingestion of food; but during the first stage of digestion, no free HCl is present—it is organically combined with proteid substances, and there are frequently present in addition traces of organic acid. During the earlier part of this stage, the small degree of acidity probably permits the amylolytic action of saliva to go on. Free HCl appears in the stomach at a variable time, between 30 minutes and the second or third hour. In second stage, both free and combined acid is present—total acidity rising. In third stage, total acidity falling. The total solids and inorganic salts

[1] *Edinb. M. J.*, July—Nov., 1893.

diminish per cent. from the commencement. Generally there is a great excess of fixed chlorides at first from the $NaCl_2$ and other chlorides swallowed with the food. Free HCl is seldom over .09 per cent. at any hour after a proteid meal, but may rise to .16 or .2 after one consisting chiefly of carbohydrates. HCl combined with proteids does not lead to heartburn, at least to any appreciable extent; but both free HCl and free organic acids do so. The pain which comes on immediately after food has been taken is probably due to the presence of an ulcer, or of erosions, or is neurotic in origin. Pain coming on at the time when free HCl should first appear in the stomach contents—allowance in calculating this must be made for the character of the foregoing meal—is probably due to this acid. If accompanied with flatulence and heartburn, and occurring at a variable time after meals, some fermentations are to be suspected, and if carbohydrates are badly borne, diminution in secretion of HCl as well.

As to treatment, deficiency of hydrochloric acid is easily remedied for a time by administering the acid by the mouth. By this means the work of the stomach is considerably lightened, and at the same time the cause of the deficiency can be treated, whether it be due to the diminution of chlorides in the blood, to increased alkalinity of the latter, to inflammation of the gastric mucous membrane, or any other of the causes of this condition.

Hyperacidity is of two classes: (1) in which HCl is in excess; (2) in which the organic acids predominate.

The first is in many cases the most difficult to deal with. Alkalies are the worst treatment for both forms of hyperacidity, as they induce the further pouring out of acid. Both ingestion of proteids and dilution of the gastric contents with water may subdue the pain, and reduction of the amount of chlorides in the food may be of service if persevered in. The excess of HCl is an expression of some nervous condition or of an abnormal state of the blood. Careful attention to the diet and general condition is needed. Frequently the liver is out of order, and then dilute nitro-hydrochloric acid and taraxacum help to improve the state of the gastric secretion.

Cases of excess of organic acids are in reality cases of deficiency of HCl, and must be treated accordingly.

If the stomach is dilated or there is chronic gastritis, lavage together with other treatment gives the best results. Gastric ulcer is accompanied by proportional HCl hyperacidity, and is to be treated accordingly. Finely divided and bland proteids by the mouth are well tolerated in many cases, the formation of proteid hydrochlorides tending to diminish pain. Milk should only be given in very small quantities. Very frequently intestinal dyspepsia arises from erroneous gastric processes, and may be cured by careful attention to the disordered gastric digestion.

Writing on diarrhœa Dr. Lauder Brunton[1] deals first with the subject of "morning diarrhœa." If we take a purgative overnight and a large draught of hot or cold water next morning, the course of events is as follows: During sleep the purgative lies in the stomach, or only proceeds some little way down the intestine; but on awaking, the movements of the alimentary canal again become brisk, and being increased by the purgative, both it and the water are hurried along through the stomach and small intestine and poured into the cæcum. They thus start increased peristaltic action in this part of the bowel, which continuing along the colon will hurry them on to the rectum. M. Chauvet thinks that in the causation of the loose motions in morning diarrhœa, the stomach-contents play a similar part to that of the purgative. In most cases the stomach is dilated, and the food instead of being expelled through the pylorus a little at a time, as it ought to be, lies in the dilated organ during sleep, and on awaking is poured out into the small intestine *en masse*, and quickly passes on to the rectum in the same way as the purgative. It is not always necessary for the patient to get up for this to occur, simple change of posture in bed may be sufficient. In Dr. Brunton's opinion, although dilatation of the stomach is present in some cases, yet morning diarrhœa is usually dependent upon an irritable condition of the sigmoid flexure or even of the rectum. Sometimes there may be only chronic inflammation or congestion; at others, actual ulceration. Unless the sigmoid flexure shares in the peristaltic movements, no action of the bowels can occur. From its letter S shape, it acts like a trap to prevent too rapid descent of the intestinal contents into the rectum; and if it remains quiet, no evacuation will take place although the rest of the intestine be in active movement. Should the sigmoid flexure, however, be irritable, and especially if ulcerated, the contents of the transverse and descending colon when poured into it will be apt to excite peristaltic movement and ejection of its contents into the rectum.

In the treatment of morning diarrhœa, all the factors that may contribute to its causation must be taken into account. For diet, entire abstinence from liquids after five, six, or seven p.m., *i.e.* for twelve or fifteen hours before the attack usually comes on, is advisable. A glass of wine may be allowed at dinner, if the patient finds this abstinence burdensome; but no soup or other liquid, and no aerated waters at night. If this limitation is insufficient to check the diarrhœa, the quantity of fluid during the earlier part of the day should be restricted, so as to lessen the amount of water in the body generally, and thus increase the rapidity of absorption. In addition, the irritability of the intestines may be lessened by the administration of such remedies as bismuth and soda, combined with carminatives and aromatics. In obstinate cases the most useful remedy is massage and kneading of the intestines.

[1] *Quart. M. J.*, Jan., 1894

For the treatment of ulceration, Dr. Brunton employs a modification of Dr. Allingham's ointment introducer, to the point of which a long rubber tube is affixed, which may be passed from 8-16 inches or more into the bowel. By turning the screw with which the instrument is furnished, and at the same time withdrawing the tube, the ointment can be applied to the bowel for a considerable distance.

Massage is also useful in that kind of diarrhœa called hill diarrhœa or sprue, to which Dr. Thin has given the name of psilosis. In this disease the best treatment, and indeed the only treatment that is likely to cure, is the exclusively milk diet advised by Sir Joseph Fayrer.

In all cases of chronic diarrhœa it is advisable never to omit an examination of the rectum, because thereby disease may be discovered in time to allow of its removal by operation.

Dr. Brunton gives a useful diet table for the treatment of such cases, the essential points in which are that all food is to be soft and finely divided, capable of passing through a hair-sieve, and that skins, bones, stringy substances, and stones of all kinds, *i.e.* in fruit, vegetables, and fish, are to be carefully avoided.

Drs. Bourneville and Paul Cornet have treated thirty cases of epilepsy by subcutaneous injections of testicular fluid,[1] and have arrived at the following conclusions:

(1) Deducting 2 cases, there remain 28 persons who were submitted to the treatment for a sufficient length of time to form a fair test of its value in epilepsy. According to d'Arsonval, if after six weeks there is no result, it is useless to persevere longer.

(2) Of the 28 cases, in 8 there was slight diminution of the fits. In the other 20 the number of fits was increased. Cases with mental failure were selected in order that a better idea might be gained of the action of the testicular fluid in promoting mental improvement. In none of them did the intellectual state show any amelioration.

(3) These results, which conform to those of M. Féré, are contradictory to those published by Prof. Pierret in a thesis by one of his pupils.

(4) In 6 cases there was a decided increase in the body-weight during treatment; in 3 others, a diminution; and in 1 it was unchanged.

(5) The injections, which were carefully carried out, gave rise to no local troubles.

J. Michell Clarke.

[1] *Progrès. méd.*, Dec. 9 and 16, 1893.

